# Sex Differences in the Heterogeneous Dynamic Incidence of Oral Cancer: A Comparison between Taiwan and Thailand

**DOI:** 10.1155/2020/9321246

**Published:** 2020-09-15

**Authors:** Pallop Siewchaisakul, Pongdech Sarakarn, Patravoot Vatanasapt, Sam Li-Sheng Chen, Amy Ming-Fang Yen

**Affiliations:** ^1^School of Dentistry, College of Oral Medicine, Taipei Medical University, Taipei, Taiwan; ^2^School of Oral Hygiene, College of Oral Medicine, Taipei Medical University, Taipei, Taiwan; ^3^Oral Health Care Research Center, College of Oral Medicine, Taipei Medical University, Taipei, Taiwan; ^4^Epidemiology and Biostatistics Department, Faculty of Public Health, Khon Kaen University, Khon Kaen, Thailand; ^5^ASEAN Cancer Epidemiology and Prevention Research Group, Faculty of Public Health, Khon Kaen University, Khon Kaen, Thailand; ^6^Khon Kaen Cancer Registry, Faculty of Medicine, Khon Kaen University, Khon Kaen, Thailand; ^7^Department of Otorhinolaryngology, Khon Kaen University, Khon Kaen, Thailand

## Abstract

**Background:**

This study aims at investigating differences in oral cancer (OC) incidence trends between two populations in Taiwan and Thailand.

**Methods:**

We used the population-based cancer registry data from Taiwan (1979-2016) and Khon Kaen (KK), Thailand (1985-2016). We performed joinpoint analyses to detect the trend change points for the OC incidence and to quantify the time trends in both sexes and regions. Age-period-cohort curves were plotted to explain the incidence trends.

**Results:**

In Taiwan, the estimated annual increases in OC were approximately 6.0% in males, although the increase slowed after 2009, and 2.6% in females. In KK, the OC incidence steadily decreased by -2.5% per year in females, but there was no obvious change in males. A strong period effect observed in those aged 45-69 years in Taiwanese males resulted in a peak incidence in the middle age group. Decreased period and cohort effects were observed in females in KK.

**Conclusions:**

Taiwanese males are the predominant sex affected by OC in Taiwan, and the trend has decelerated since 2009. Age, period, and cohort effects were different between males and females in the two regions.

## 1. Introduction

Although the oral cancer (OC) burden is not the highest compared with other common cancers, such as colorectal cancer and breast cancer, the disease pattern is characterized by great regional heterogeneity. The highest estimated number of OC cases worldwide in 2018 occurred in Asia [1]. Considerable heterogeneity with respect to demographics and the dynamic incidence rate of OC is observed, even within Asian regions. For example, Taiwan has a high OC incidence rate with male predominance (age − standardized incidence rates (ASIRs) = 62.50 and 12.86 per 100,000 in males and females, respectively) [2], whereas the trend is opposite in the Khon Kaen Province, Thailand (ASIRs = 3.1 and 2.9 per 100,000 in females and males, respectively) [3].

Regarding the time trends of the OC incidence in both sexes, previous studies in Taiwan revealed that there was a dramatically increasing trend in OC from 2002 to 2012 in males (a significant estimated average annual percent change (EAAPC) of 6.7%) but not in females (EAAPC of 2.0%) [2]. However, females had a higher incidence of OC in some regions of Thailand, such as Khon Kaen, than males [4].

Three risk factors including betel quid (BQ), smoking, and alcohol consumption have been established as etiologies of OC. The demographic and geographical heterogeneity in the time trends of the OC incidence is thought to be explained by different consumption levels of the three risk factors according to sex and age, with different exposure levels to these risk factors in different calendar years. This can also account for the heterogeneous time trends in Thailand [5], in which a previous study found that BQ use was more common in females than in males in Khon Kaen [6].

As exposure to the three risk factors in each region which is subject to the exposure opportunity, the three time-related dimensions of age, period, and birth cohort (APC) [7] play an important role in elucidating the mechanism of heterogeneity in the disease burden of OC. Age effects normally account for deterioration in the immune system activity; however, they can also represent the exposure duration to the three risk factors, which can cause epigenetic and genetic damage to the DNA over the lifespan. Period effects account for the results of external factors, such as intervention policies affecting primary prevention and secondary prevention, or new reporting systems that equally influence all age groups during the study period. The latter is popular, especially in Asian countries, such as Taiwan and Thailand [8]. Currently, human papillomavirus (HPV) is considered to be a cause of OC and oropharyngeal cancer (OPC) [9], and several studies have shown HPV to contribute to OPC incidence trends [10–12]. In the United States, an increasing incidence rate was observed in consecutive birth cohorts in males, and the increase was postulated as being caused by increased exposure to oral HPV in recent birth cohorts [13]. The identification of APC effects from regions with heterogeneous disease patterns would be helpful for elucidating the change mechanisms.

Although the time trends of the OC incidence considering APC effects have been studied in a variety of cancers, these effects have rarely been applied to exploring the time trends of OC. In addition, no published study applies APC curves to OC in Taiwan and Khon Kaen, Thailand. It is of interest to compare the trends of the OC incidence between Taiwan and Khon Kaen, Thailand, where the epidemiological disease profiles differ greatly with respect to sex and time. This study aims at using APC curves to explore and compare the time trends of the OC incidence relevant to APCs between Taiwan and Thailand.

## 2. Methods

### 2.1. Data Source

Information regarding all new OC cases in Taiwan, namely, the OC incidence data from 1979 to 2016, were obtained from the Cancer Registry of the Health Promotion Administration, Ministry of Health and Welfare. In Thailand, data were retrieved from the population-based cancer registry of Khon Kaen Province for all cases diagnosed between 1985 and 2016. OC cases were determined according to the International Classification of Diseases for Oncology, 3rd edition, codes C00, C01-C02, C03, C04, C05, C06, C9-C10, C12-C13, and C14.

### 2.2. Statistical Analysis

We first examined the sex-specific ASIRs over time in both regions. The ASIRs, considering the world standard population in 2000, were directly calculated, with thirteen age-specified groups; the youngest was 20 to 24 years, and the oldest was 80 to 84 years. We excluded those diagnosed at age < 20 and over 84 years due to the rarity of cases. Chronological changes in the OC trends in both Taiwan and Khon Kaen were analyzed using a joinpoint regression model. The estimated annual percent change (EAPC) and EAAPC were weighted for the entire interval. All calculations were considered statistically significant when the *P* value <0.05 [14].

We categorized patient age into thirteen 5-year groups (20 to 24,…, 75 to 79, and 80 to 84 years) in both regions, we categorized period into seven 5-year intervals for Taiwan (1982 to 1986,…, 2007 to 20011, and 2012 to 2016) and Khon Kaen (1987 to 1991,…, 2007 to 2011, and 2012 to 2016), and we categorized birth cohorts into 5-year intervals in Taiwan (starting with 1898 to 1902 until 1988 to 1992) and Khon Kaen (starting with 1903 to 1907 until 1988 to 1992). To explore the effects of APCs on OC incidence trends, we plotted APC curves. Data for the APC curves were managed and analyzed using Stata (StataCorp. 2007. Stata Statistical Software: Release 10. College Station, TX: StataCorp LP).

### 2.3. Ethical Considerations

The use of Thailand's data for this study was approved by the Khon Kaen University Ethics Committee for Human Research (reference number: HE611129). There was no requirement for the use of Taiwan's data, as they are open access.

## 3. Results

### 3.1. Descriptive Results

Tables 1 and 2 show the annual incidence of OC by sex, age, and period in Taiwan and Thailand. In total, there were 125,167 OCC incident cases for both sexes (114,195 for males and 10,972 for females) between 1979 and 2016 in Taiwan. In Khon Kaen, the number of incident cases of OCC in both sexes was 1,752 (647 for males and 1,105 for females) during1985-2016. The sex ratio (male to female) was 10.4 : 1 in Taiwan, but the reverse was noted in Khon Kaen (0.6 : 1). The marked different incidence rate in Taiwan males was at aged 55-59 between 1982-1986 (16.93/100,000) and 2012-2016 (144.27/100,000), while in Taiwan females was found at aged 75-79 between 1982-1986 (4.12/100,000) and 2007-2011 (19.01/100,000). In Thailand, we found marked difference of an incidence rate which was found in elderly at aged 80-84 between 1992-1996 (77.46/100,000) and 2012-2016 (30.99/100,000) for male and was at aged 70-74 between 1987-1981 (68.59/100,000) and 2012-2016 (16.36/100,000) for female.

The incidence in Taiwan increased with both age and period in females. However, in males, the incidence increased with period but not with age; in fact, the incidence started to decrease after the age of 64. Nevertheless, an increasing trend with age existed in all male birth cohorts. In Thailand, the incidences in both sexes varied with age and period. However, decreasing trends were observed in elderly males and females.

### 3.2. The Trends of ASIRs of OC

**Figure 1:**
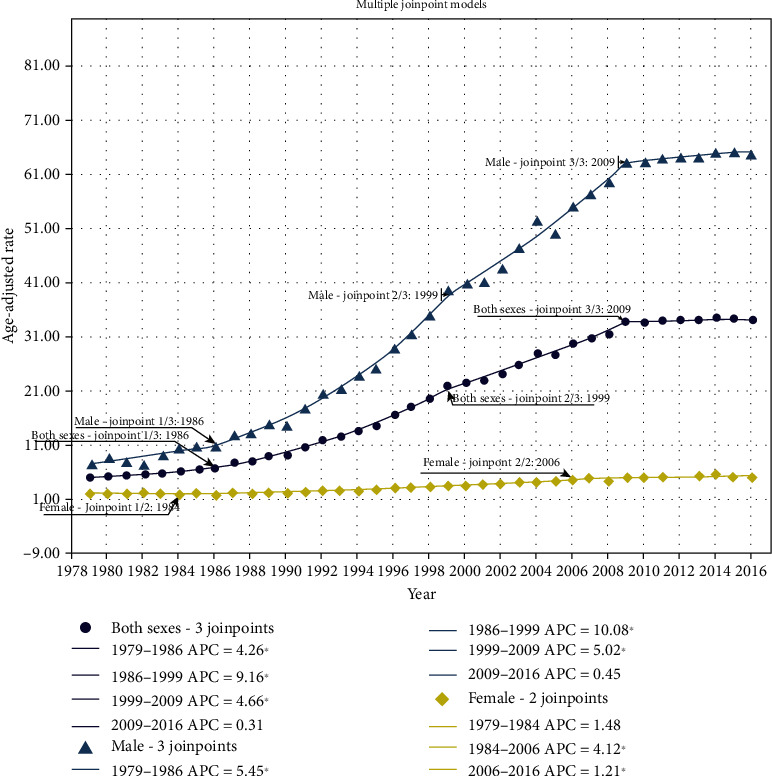
Age-standardized incidence rates (per 100,000) of oral cancer by sex in Taiwan, 1979-2016.

Between 1979 and 2016, the incidence of OC in Taiwanese males dramatically increased with time and predominated over females (Figure 1). Three significant change points were detected in males in 1986 (EAPC = 5.5%; 95% CI: 2.3, 8.7), 1999 (EAPC = 10.1%; 95% CI: 9.2, 10.9), and 2009 (EAPC = 5.0%; 95% CI: 4.3, 5.7). As males had a higher incidence of OC than females in Taiwan, males dominated the total population (both sexes) curve, with EAPCs of 4.3% (95% CI: 1.4, 7.2), 9.2% (95% CI: 8.3, 10.0), and 4.7% (95% CI: 4.0, 5.3), respectively. In females, two significant change points were observed in 1984 (EAPC = 4.1%; 95% CI: 3.6, 4.7) and in 2006 (EAPC = 1.2%; 95% CI: 0.2, 2.2). The EAAPC (full range) was 5.3% (95% CI: 4.7, 5.9), 6.0% (95% CI: 5.3, 6.6), and 2.6% (95% CI: 1.5, 3.6) for the total population, males and females, respectively.

**Figure 2:**
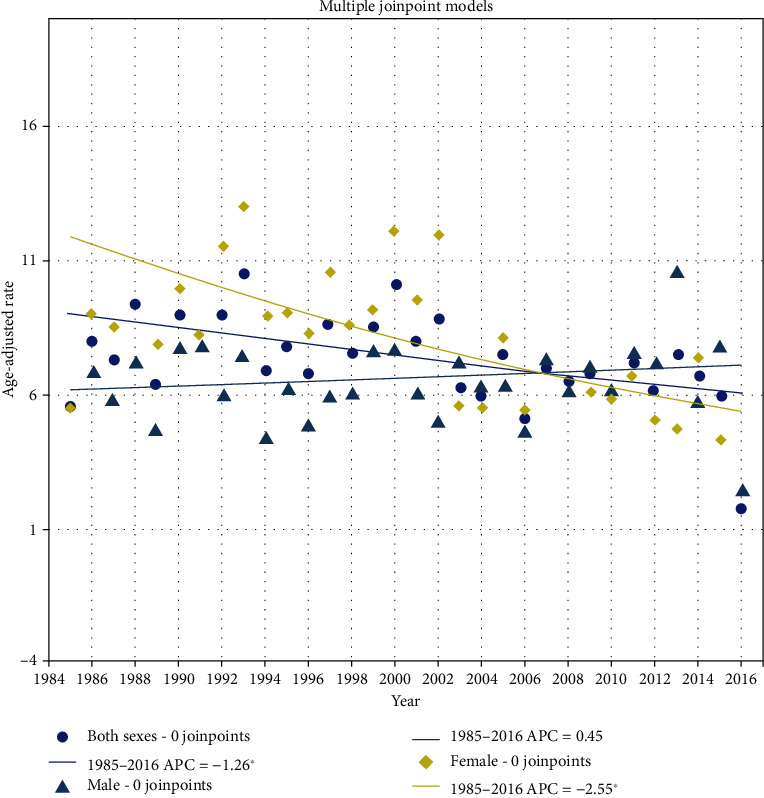
Age-standardized incidence rates (per 100,000) of oral cancer by sex in Thailand, 1985-2016.

In contrast to Taiwan, the OC trends showed no significant change points in either males or females in Khon Kaen. Gradually, decreasing trends were observed in females (EAPC = −2.5%; 95% CI: -3.7, -1.4) and in the total population (EAPC = −1.3%; 95% CI: -2.1, -0.4). However, trends steadily increased, with nonsignificant differences, in males (EAPC = 0.4%; 95% CI: -0.6, -1.5) (Figure 2). Females had higher incidence rates of OC in the 1980s than males, but this pattern was reversed in the 2010s. The EAAPC values in Thailand were the same as the EAPC values.

### 3.3. The Age, Period, and Cohort (APC) Curves of the OC Incidences

**Figure 3:**
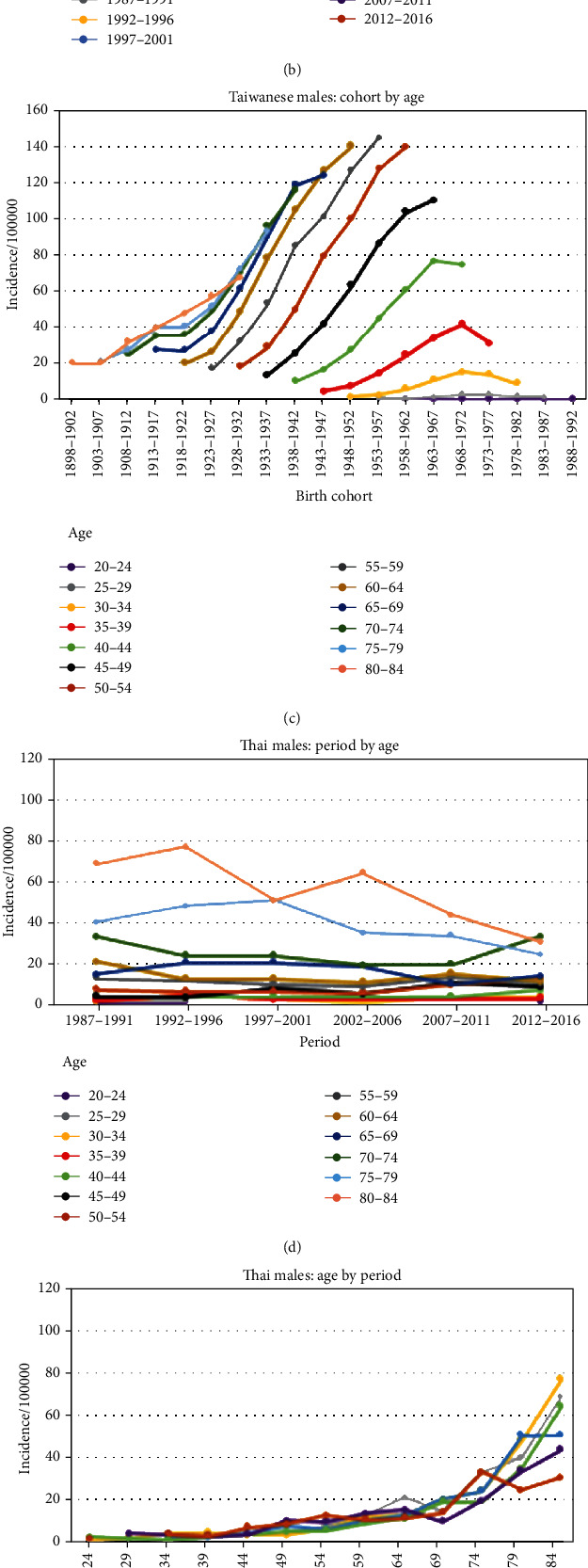
APC curves for males in Taiwan (1982-2016) and Thailand (1987-2016).

As shown in Figure 3(a), the OC incidences in Taiwanese males increased with period in most age groups except for the 20-29 years age group. There was a tremendous difference in the incidence rates between the 30-34 and over 40 years age groups in the period 1992-1996 and a large difference in the incidence in the 50-54 years age group (127.34 per 100,000) between 1982-1986 and 2012-2016. As shown in Figure 3(b), since 1997, the incidence rates dramatically increased with age until the age of 50, and after the age of 64, they declined. As shown in Figure 3(c), the incidence rates increased in consecutive cohorts in most age groups, and the incidence was higher in the older age group than in the younger age groups in the same birth cohort.

In males in Khon Kaen, the high incidence rates were found among those over 60 years of age, with the highest incidence rates occurring in those 80 to 84 years of age in 1992 and 1996 (77.46 per 100,000). A decreasing trend was observed in the older age (over 60) group, while age groups younger than 55 years showed a modestly increasing trend. In the past, tremendous gaps between age groups older and younger than 75 years have been observed. Nevertheless, the gap narrowed in the most recent period, as shown in Figure 3(d).  Figure 3(e) shows an incremental gap in the OC incidence that was driven by age because the incidence rates increased with age in the same period and birth cohorts. The cohort curve by age in Figure 3(f) shows a decreasing incidence in the recent birth cohort, especially for those aged over 60 years.

**Figure 4:**
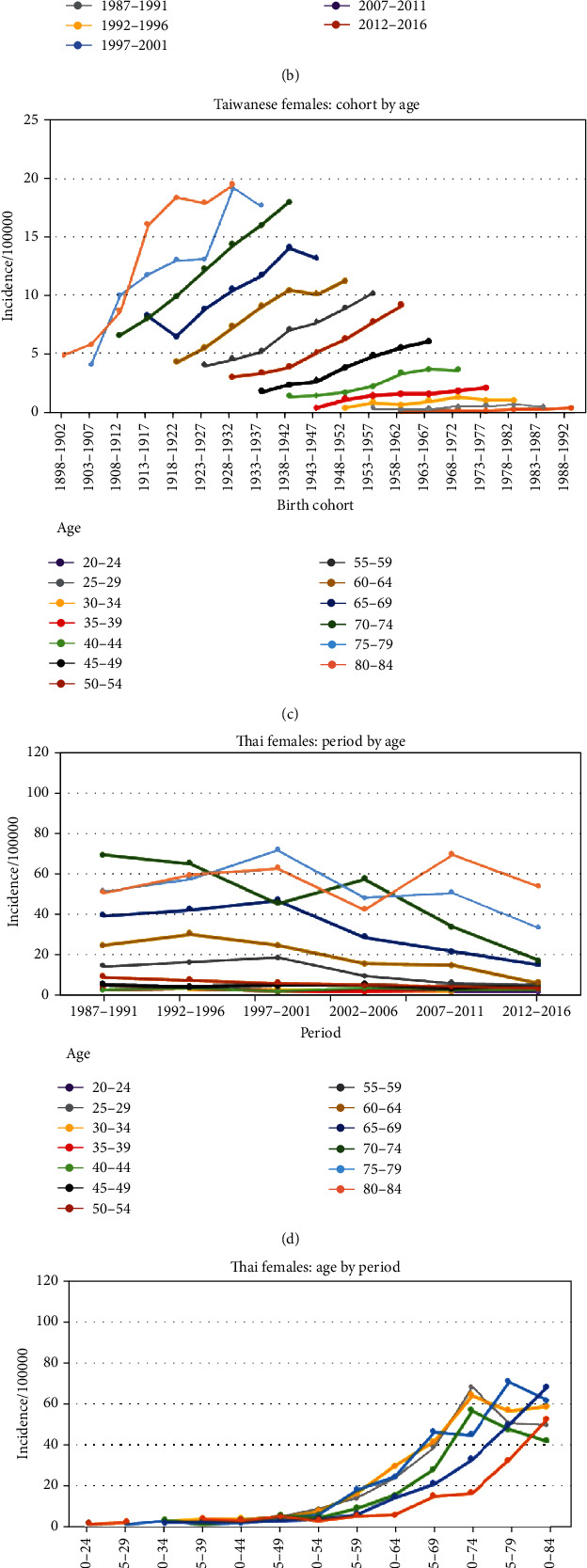
APC curves for females in Taiwan (1982-2016) and Thailand (1987-2016).

The time trends of the OC incidence in females in Taiwan and Thailand according to APC are shown in Figure 4. As shown in Figure 4(a), the OC incidence in Taiwanese females increased with period in most age groups and modestly increased in the age groups younger than 40 years. Unlike Taiwanese males, we observe that older individuals had a higher incidence than younger individuals in the same period, for example, in the 2007-2011 period. As shown in Figure 4(b), the increase in the incidence in most of the periods was driven by age; however, the incidence rates in the age groups 65-69 and 75-79 years were found to drop in the periods of 1982-1986 and 1987-1991, respectively. The incidence trends for females stratified by age group were the same as those in males, all of which increased in the most recent cohort in the same age group and were higher than the incidence trends by age in the same birth cohort (Figure 4(c)).

The highest incidence of OC in Thai females was 68.7 per 100,000 (age 80-84) in the period of 2007-2011 (Table 1). The incidence in almost all of the age groups among Thai females showed decreasing trends with periods, and only the age group of 80-84 showed a zigzagging trend (Figure 4(d)). As seen in Figures 4(e) and 4(f), the incidence trend was also mainly driven by age. For example, analyzing the same age group of 70-74 years in different periods showed that the earlier the period occurred, the higher the incidence was; this pattern was quite consistent. A large difference was observed between the birth cohorts born before and after 1928-1932 beginning in the 65-69 age group.

## 4. Discussion

This study was the first to compare the OC incidence rates in Taiwan and Khon Kaen, Thailand; there were some distinct characteristics between the two regions, including the dominant sex (male in Taiwan and female in Khon Kaen), opposing trends (increasing in Taiwan and decreasing in Khon Kaen), and cross-sectional age-specific incidence patterns (concave in Taiwanese male since 1997).

The differences and changes in the OC incidence patterns could be explained by the three established risk factors of chewing BQ, smoking cigarettes, and drinking alcohol [15]. BQ chewing has long been known to be the most significant risk factor for OC in Taiwan and Thailand. In Taiwan, the estimated number of habitual BQ chewers was 2 million (10%) among the whole population [16], and BQ chewers were more likely to be male than female. BQ chewing peaked in the 1980s, and consumption increased 10-fold from 1992 to 2011. Areca nut cultivation expanded considerably, with a 58-fold increase in the number of plants grown from 1981 to 1990, while only 4% of its production was for export [17].

The increased tendency in BQ chewing, as described above, supports the increased OC incidence over time, as well as the notable EAPC from 1986 to 1999 in males. Remarkably, we observed that the increase in the trend started to decelerate between 2009 and 2016 (Figure 1), which may have resulted from the recent anti-BQ campaign by the Taiwanese government. The prevalence of BQ chewing has begun to decrease in recent years [18]. Another possible reason is the nationwide oral screening program that was initiated in 2004 in Taiwan; the program targets oral potentially malignant disorders (OPMDs) and OC in subjects who habitually smoke or chew BQ [19].

The BQ chewing prevalence in Thailand was notably different from that in Taiwan. Thai females had a higher prevalence than Thai males. A recent study showed that the prevalence of BQ chewing was approximately 1.8% in the total population and 0.3% and 3.3% in males and females [20], respectively. This is also consistent with a study in Khon Kaen from 1990-2001, in which the authors found a large difference in the prevalence between females (24.0%) and males (1.2%) [6]; however, formerly, in the rural northern Thailand, the prevalence in males was 16%; this former rate in Thai males is comparable to the current rate in Taiwanese males [21]. The use of BQ is drastically declining because the Thai government established a policy in 1940 to prompt citizens to quit chewing BQ and ordered the elimination of all betel trees throughout the country. The first observed potential decline in BQ chewing occurred in 1955 [22]. Consequently, we observed a high incidence of BQ chewing in only the elderly age groups.

Age is one of the most common risk factors in epidemiological studies. Cancers are known to be age-related, and the risk of cancer increases with age [23]. There were inconsistencies in our APC curves since the curves for Taiwanese males depicted the peak incidence at 50 years of age and a declining trend after 64 years of age in most periods (Figure 3(b)). The peak incidence at age 50 might be explained by the latency periods of OPC and other OCs, which were 12.3 years and 16.9 years, respectively [24]. Therefore, it is possible that someone who is exposed to risk factors at age 35 will be diagnosed with cancer at approximately age 50. This hypothesis was supported by the most common age of BQ chewing onset (adolescent students) [25], at 23 to 35 years old, with a prevalence of 23.9% in males and 2.0% in females [26]. We believe that the relatively low incidence in the elderly age groups is because BQ chewing was not popular when these individuals were in their 30s. Conversely, in Taiwanese females, the prevalence of BQ chewing increased with age [27]. This sex disparity occurred because the majority of Taiwanese female chewers were indigenous individuals, and BQ chewing is part of their culture. Accordingly, BQ chewing has not been dramatically affected by the increase in areca nut cultivation since 1981.

The smoking rates in males in both countries were similar and showed a continuously decreasing trend. The prevalence of smoking in males in Taiwan and Thailand was 40.5% between 2001 and 2013 and 42.1% between 2001 and 2011, respectively [28, 29]. This trend was consistent with that in Thai females (2.5%) [30]. However, in Taiwanese females, the prevalence of smoking was approximately double that of Thai females and showed an increasing trend from 2001 to 2009 [28]. Therefore, cigarette smoking might play an important role in the increasing OC trend in Taiwanese females.

In Thailand, an almost BQ-free country, people still have a unique type of roll-your-own (RYO) cigarette called Yamuan. This kind of cigarette is possibly more dangerous than factory-made cigarettes [31]. A study in Khon Kaen from 1990 to 2001 revealed that 62% of current smokers smoked only Yamuan [32]. This might be the reason why we observed a nonsignificant increase in the incidence in Thai males, as shown in Figure 2. However, smoking seems to be a minor factor in OC incidence in Thai males and females because the trends in smoking prevalence decreased in both sexes and were minimal, especially among females.

In Taiwan, alcohol consumption was concluded to play a minor role in the development of OPMDs and thus OC in previous studies [33]. In Thailand, a study revealed a significant increase in the OC risk with alcohol consumption among females but not among males [34]. As a result, alcohol consumption in Thailand could be considered a contributing factor to the increasing incidence of OC in males.

We found that the incidence in Taiwan increased in the recent cohort in all age groups. One reason for this may be that the period effect was probably occurred by the birth cohort effect because the same pattern appeared in the different age groups. Unlike the pattern that is shown in Figure 3(f) in Thai males, the age groups over 60 showed a decrease in the incidence in the recent cohorts, whereas those of the age groups younger than 60 remained stable. Therefore, the birth cohort effect was expected; unfortunately, it was not clearly observed. However, we also observed an increasing trend from 2000 to 2016 in male Thai individuals younger than 34 years old. Currently, HPV is considered a cause of OC. In the United States, an increase in the incidence rate was observed in consecutive male birth cohorts; this increase was postulated to be due to increased exposure to oral HPV in recent birth cohorts [9]. Whether the increasing trend in OC in younger age groups in Asia is associated with HPV needs further investigation.

In this study, we explored the differences in oral cancer (OC) incidence trends by sex, period, and birth cohort, between two populations in Taiwan and Thailand, and therefore, we cannot look into detailed information by the anatomic site due to sparse cases in Thailand. Moreover, we cannot clearly elucidate specific etiologies, especially for OC cases at the base of the tongue and in the anterior two-thirds of the tongue, which are considered to have distinct etiologies. A further study with different methodology which can tackle the issue of sparse sample size is needed.

In conclusion, an increasing trend in the OC incidence was observed in Taiwan, but the increase began to decelerate in 2009. APC effects were different in males and females in both regions, which may have significant implications for different intervention policies and the use or consumption of high-risk substances over with time in the two regions.

## Figures and Tables

**Table 1:** Incidence rates (per 100,000) by age, sex, and period in Taiwan and Thailand.

Age group	Taiwan						
	1982-1986	1987-1991	1992-1996	1997-2001	2002-2006	2007-2011	2012-2016
Male							
20-24	0.20	0.33	0.58	0.62	0.39	0.45	0.48
25-29	0.69	1.13	1.73	3.27	3.40	2.32	1.87
30-34	1.86	3.09	6.51	10.99	15.60	13.97	9.17
35-39	5.04	7.71	14.71	25.14	34.28	41.67	31.39
40-44	10.48	16.57	27.63	45.19	60.53	76.64	74.20
45-49	13.43	25.29	41.87	63.30	86.34	103.40	109.76
50-54	18.29	28.65	49.74	79.42	100.00	126.67	139.03
55-59	16.93	31.62	53.43	84.87	101.15	126.31	144.27
60-64	20.04	26.18	48.49	78.44	104.38	125.97	139.72
65-69	27.26	26.94	38.17	61.29	90.25	117.88	123.56
70-74	24.91	35.26	35.73	49.28	70.26	95.55	115.07
75-79	21.25	27.56	40.34	40.65	52.14	72.70	93.01
80-84	20.04	19.89	31.93	40.03	47.80	57.25	67.79

Female							
20-24	0.13	0.27	0.25	0.18	0.35	0.41	0.46
25-29	0.40	0.35	0.39	0.59	0.67	0.73	0.58
30-34	0.45	0.89	0.75	1.03	1.38	1.12	1.13
35-39	0.49	1.16	1.52	1.61	1.66	1.90	2.13
40-44	1.44	1.54	1.78	2.27	3.44	3.73	3.65
45-49	1.87	2.39	2.73	3.87	4.85	5.58	6.12
50-54	3.09	3.39	3.90	5.16	6.31	7.70	9.19
55-59	4.06	4.58	5.26	7.09	7.71	8.89	10.16
60-64	4.34	5.59	7.35	9.00	10.41	10.09	11.20
65-69	8.22	6.45	8.81	10.49	11.62	14.00	13.06
70-74	6.58	8.02	9.86	12.19	14.24	15.89	17.83
75-79	4.12	10.01	11.74	12.97	12.98	19.01	17.50
80-84	4.88	5.81	8.63	15.98	18.27	17.81	19.34

**Table 2:** Incidence rates (per 100,000) by age, sex, and period in Thailand.

Age group	Thailand					
	1987-1991	1992-1996	1997-2001	2002-2006	2007-2011	2012-2016
Male						
20-24	1.24	1.30	NA	2.84	NA	1.34
25-29	2.19	2.15	NA	1.73	4.17	NA
30-34	1.57	4.43	2.67	1.52	3.74	4.01
35-39	2.41	4.63	2.45	2.94	3.09	3.15
40-44	4.09	3.65	4.16	3.82	3.89	7.15
45-49	3.92	3.58	7.83	5.16	10.50	8.64
50-54	7.66	6.52	6.48	5.60	9.76	12.78
55-59	12.71	11.92	10.17	8.90	13.74	10.68
60-64	21.34	12.91	12.72	11.07	15.25	11.66
65-69	14.82	20.56	20.50	19.12	10.06	14.01
70-74	33.51	24.26	24.39	19.28	19.59	33.51
75-79	40.36	48.49	50.84	35.30	33.93	24.67
80-84	69.25	77.46	51.00	64.76	44.05	30.99

Female						
20-24	NA	NA	NA	NA	1.57	1.49
25-29	1.87	NA	1.34	NA	NA	2.21
30-34	NA	2.37	2.52	2.68	1.58	NA
35-39	2.58	3.28	1.40	1.40	2.13	3.68
40-44	2.11	3.46	1.56	2.79	2.03	2.68
45-49	4.93	3.82	4.52	4.91	2.85	4.87
50-54	8.51	7.23	5.36	4.34	3.65	2.91
55-59	13.67	15.88	17.88	8.81	5.67	4.93
60-64	23.97	29.52	24.17	15.10	14.12	5.43
65-69	38.83	41.57	46.32	28.15	20.88	14.76
70-74	68.59	64.35	44.78	56.82	33.02	16.36
75-79	50.75	56.87	71.11	47.51	49.90	32.60
80-84	50.00	58.83	61.97	41.79	68.70	52.96

## Data Availability

No additional data are available, and the data from the cancer registry in Khon Kaen, Thailand, require ethical proof to access.
